# Isolation and characterization of microsatellite loci for *Prunus mongolica* (Rosaceae)

**DOI:** 10.1002/aps3.1158

**Published:** 2018-06-26

**Authors:** Yu‐Chen Cheng, De‐Jian Zhang, Zhan‐Yuan Lu, Xue‐Song Ye, Jian‐Guo Wang, Ping Sun, Bao‐Wei Zhang

**Affiliations:** ^1^ Inner Mongolia Academy of Agriculture and Animal Husbandry Sciences Hohhot Inner Mongolia Autonomous Region China; ^2^ School of Life Sciences Inner Mongolia University Hohhot Inner Mongolia Autonomous Region China; ^3^ School of Life Sciences Anhui University Hefei Anhui Province China

**Keywords:** genetic diversity, microsatellite, polymorphism, *Prunus mongolica*, Rosaceae

## Abstract

**Premise of the Study:**

Microsatellite primers were developed in *Prunus mongolica* (Rosaceae), a relict flora endemic in arid areas of the Asian interior, to investigate the genetic diversity, phylogeography, population structure, and history of the species.

**Methods and Results:**

Fifty‐one microsatellite loci, including di‐, tri‐, and tetranucelotide repeats, were identified using transcriptome sequencing and bioinformatic screening. The number of alleles ranged from seven to 11 and the levels of observed and expected heterozygosity ranged from 0.545 to 1.000 and 0.600 to 0.989, respectively. Most of the primers also amplified in a group of congeneric species (*P. triloba*,* P. davidiana*,* P. persica*,* P. cerasifera*, and *P. serrulata*).

**Conclusions:**

This set of microsatellite loci is useful for studying the genetic diversity of *P. mongolica*. In addition, they can also be used to investigate the population structure, phylogeography, and landscape genetic patterns of congeneric species.

*Prunus mongolica* Maxim. (Rosaceae) is a relict flora that is endemic to arid areas of the Asian interior. *Prunus mongolica* was listed in the China Plant Red Data Book as endangered; this has resulted from mining, firewood collection, industrialization, and urbanization, which have led to habitat loss and fragmentation (Fu, 1992). Scientific conservation requires a full understanding of genetic diversity, phylogeny, and population structure. Genetic studies are limited in *P. mongolica*; therefore, it is necessary to develop microsatellite loci for use in the species.

Microsatellites, also known as simple sequence repeats (SSRs), have been widely used as DNA markers in studies of population genetics, phylogeography, landscape genetics, and parentage analysis due to their high level of polymorphism, putative neutrality, codominant inheritance, and ease of scoring (Moriguchi et al., 2015). In this study, we isolated and characterized 51 polymorphic microsatellite loci for *P. mongolica* by transcriptome sequencing and bioinformatic screening.

## METHODS AND RESULTS

Total RNA was extracted from fresh leaves of one individual of *P. mongolica* using the EasyPure Plant RNA Kit (TransGen Biotech Inc., Beijing, China) following the manufacturer's protocol. A cDNA library was prepared using the TruSeq Stranded Total RNA Sample Prep Kit (Illumina, San Diego, California, USA) and sequenced on a HiSeq 3000 sequencing platform (Illumina).

Extra (adapters and other Illumina‐specific sequences) and low‐quality sequences were removed from the raw data using the software Trim Galore version 0.4.4 (Li et al., 2015). FastQC version 0.11.5 software (Yang et al., 2013) was used to analyze the quality control of the pre‐processed data. A total of 53,866 contigs were obtained by de novo assembly using Trinity version 2.3.2 (Grabherr et al., 2011). SSR detection was performed with MISA version 1.0 (Thiel et al., 2003) with the following criteria: ≥6 repeat units for dinucleotides, ≥5 for tri‐, and ≥4 for tetra‐, penta‐, and hexanucleotides. Primer sets were designed with Primer3 using the default parameters (Koressaar and Remm, 2007; Untergasser et al., 2012). A total of 19,498 SSRs were identified from 15,837 contigs, and 6069 primer sets were designed. Two hundred primer sets that amplified di‐, tri‐, and tetranucelotide repeats with ≥6, ≥5, and ≥4 repeat units, respectively, were selected to test for polymorphism. Raw transcriptome data were deposited in the National Center for Biotechnology Information Short Read Archive (BioProject no. PRJNA418876, BioSample no. SAMN08038438).

Because of habitat loss, only 32 *P. mongolica* individuals were sampled from three populations (Appendix 1). Total genomic DNA was extracted from 0.15 mg of leaf tissue using a modified cetyltrimethylammonium bromide (CTAB) method (Wang et al., 2010). All PCRs were performed in total volumes of 15 μL: 1 μL of DNA, 5.5 μL of sterilized deionized water, 7.5 μL of 2× EasyTaq PCR Supermix (TransGen Biotech Inc.), and 0.5 μL each of forward and reverse primers (each forward primer was fluorescently labeled with FAM, HEX, or TAMRA). PCR amplification was carried out using the following thermal conditions: initial denaturation for 5 min at 94°C; then 35 cycles of denaturation for 30 s at 94°C, annealing for 30 s at the annealing temperature (Table 1), and 20 s at 72°C; followed by a final extension for 10 min at 72°C. PCR products were genotyped on an ABI PRISM 3730 genetic analyzer (Applied Biosystems, Waltham, Massachusetts, USA) with a GeneScan 500 size standard (Applied Biosystems). Allele peaks were scored using GeneMarker version 1.3 (SoftGenetics, State College, Pennsylvania, USA). MICRO‐CHECKER version 2.2.3 (van Oosterhout et al., 2004) was used to detect genotyping errors due to null alleles, stuttering, or allele dropout. The number of effective alleles and levels of observed and expected heterozygosity were calculated using GENETIX version 4.0 (Belkhir et al., 2001). Deviation from Hardy–Weinberg equilibrium and linkage disequilibrium was tested with GENEPOP version 3.4 (Rousset, 2008).

**Table 1:** Characteristics of 51 microsatellite loci developed in *Prunus mongolica*

Locus	Primer sequences (5′–3′)	Repeat motif	Allele size range (bp)	*T*_a_ (°C)	Function	GenBank accession no.
TR3320	F: CGCCTCCTCTCTCTCTCTA	(TC)_19_	127–175	55	EIN3‐binding F‐box protein 1	MG682092
	R: CCCAGAAAAGATTAAGGCTAC					
TR5622	F: AAACGAGAGCAGGATCAATA	(CAA)_8_	127–179	55	B3 domain‐containing transcription factor	MG682093
	R: CGTAGTTTGGGTTGGAGTC					
TR9296	F: GCAGAGAACTTATCGCTTATG	(TTTA)_6_	133–201	55	Glyoxylate/hydroxypyruvate reductase HPR3	MG682094
	R: CATCTGGAAAGTGAATCTGAG					
TR10339	F: ACTCTTCTCTCCATTGAGTCC	(CT)_20_	105–141	55	Protein MIZU‐KUSSEI 1	MG682095
	R: GTCCTCATCTGGGAAATTTAG					
TR10936	F: AGAAGATAGAGGGATGGGAGT	(AG)_21_	153–181	56	GATA transcription factor 11	MG682096
	R: CTCTCTGCAACAACCAAAAC					
TR12256	F: GAGAGAGAAAAATTGCAGTCC	(TCT)_7_	131–177	55	Unknown	MG682097
	R: CCATGGAGATTAAGAGGAAGA					
TR12298	F: CAATAGCCAAAACCCTCTC	(TC)_6_	123–169	54	DNA‐directed RNA polymerases II, IV, and V	MG682098
	R: GCCTTCCATTTCTCTATGTCT					
TR12728	F: AAATCTAAGCCCACGTACATC	(GA)_18_	153–193	56	Protein‐tyrosine‐phosphatase MKP1	MG682099
	R: CACCCTGGTAACTCATAATCA					
TR14394	F: TGAGCTACAGACAGGTGAACT	(GAA)_7_	143–169	55	Transcription factor bHLH25	MG682100
	R: ACACAACACACCACTTTTCTC					
TR16484	F: GTGTTGGCTAGAGTGACTTTG	(TC)_8_	157–195	56	UDP‐glycosyltransferase 74F2	MG682101
	R: CGCCACACCTAAGTTTTT					
TR17663	F: TCATCTCCTCTACCTTTTTCC	(CT)_19_	115–169	55	Unknown	MG682102
	R: GGTCATGATCTCTGCTTGATA					
TR17745	F: CCTTCCATTTCTCTGTTCTCT	(TC)_21_	111–149	55	Aquaporin PIP1‐2‐like	MG682103
	R: CACTCAACCAAATCATAGCTC					
TR18640	F: AACTAGAGAGAGAGACGCACA	(GA)_16_	91–139	55	Succinate‐CoA ligase [ADP‐forming] subunit beta, mitochondrial	MG682104
	R: GTTCTTTCGCATTCTCAGAC					
TR18685	F: CAGAAGTCAGAGCAGAGTCAG	(ACAG)_6_	139–169	55	VQ motif‐containing protein 9	MG682105
	R: CTACCGGGAGATATTTCAGAT					
TR18807	F: GTCCTTAAGCAGCCCAGTA	(CT)_20_	121–173	55	Probable acyl‐activating enzyme 17, peroxisomal	MG682106
	R: AGAGCTTCGATGTCAGAGAC					
TR19174	F: CTTGAGAGGAGGAAAAAGAAG	(AGA)_8_	145–217	55	Transcription factor PRE6‐like	MG682107
	R: GCTGTCTGGGGTTATAAATTC					
TR19201	F: TATTGCACTCTCCTATGAACC	(CAG)_7_	139–167	55	Auxin response factor 7 (LOC110749255), transcript variant X2	MG682108
	R: GTTGAGACTCTTGCTGTTGTT					
TR20374	F: CTTCCATCCACACTTACAT	(GA)_8_	149–207	55	Uncharacterized protein At1g04910	MG682109
	R: GAGGGACTGAATAAACCTGAG					
TR21328	F: AAGGTGAAGGGTGAAGATACT	(GA)_12_	141–195	55	Magnesium‐chelatase subunit ChlH, chloroplastic	MG682110
	R: AAACACAATCACAGAGAGAGC					
TR21918	F: CCTCCTTCTGTCTCTCTCTTC	(CT)_12_	125–187	55	E3 ubiquitin‐protein ligase RNF144A	MG682111
	R: CAGTTAAACCACCAGCACTAC					
TR20030	F: ATAACCCCAAGCTCTCTCTC	(CTC)_8_	131–161	55	*Prunus persica* receptor‐like protein 51	MG682112
	R: AGGAGGTAGGAGATGGAGTG					
TR22071	F: CAGAAGAAAAAGAAGGAGAGC	(GAA)_7_	131–161	55	*Prunus avium* cysteine proteinase inhibitor 12‐like, transcript variant X6	MG682113
	R: TGTCAACCATACTCACCTCAT					
TR23600	F: CAGCTCTCTCTTTCTCTCTCC	(TC)_8_	141–183	55	Probable serine/threonine‐protein kinase DDB_G0282963	MG682114
	R: ACTTCCACTACCAGAATCCAT					
TR24122	F: GGTCTGTTAATGGAGGTTCTT	(CT)_13_	133–169	56	UDP‐*N*‐acetylglucosamine–dolichyl‐phosphate	MG682115
	R: CACCAATATTGAGAGAGAGAC					
TR25043	F: AACCAACCCAACCCACAC	(ACAT)_6_	111–165	57	*N*‐acetylglucosaminephosphotransferase	MG682116
	R: GGGGGAGAACCTATCTTATATT					
TR25166	F: CAGGCTCTGTAGTTCGATTC	(TCT)_8_	147–179	55	Gibberellin 20 oxidase 1	MG682117
	R: TGTATGTTAGTAAGGCGAAGG					
TR25414	F: CAGCAGAGAGACCAGAATAGA	(TCCA)_6_	103–125	55	Uncharacterized protein At2g17340‐like	MG682118
	R: GAGCATTATAGCTGTGTGTGG					
TR26908	F: TATCTTCATCGCTCTCTTCTG	(CAT)_10_	111–149	55	Galactomannan galactosyltransferase 1	MG682119
	R: CTGGGGTCTTAATTAGACGTT					
TR27527	F: CACCAAACCTTTCTCTCTCTT	(CT)_27_	117–165	54	Transcription factor HY5‐like, transcript variant X1	MG682120
	R: ACAAAGTCGATCAGCATCTC					
TR29327	F: CTGCCGTCTATCTCTCTCTCT	(CT)_6_	113–145	56	Probable fructokinase‐4	MG682121
	R: CAAGTTTGGTGCTCGTATCTA					
TR30962	F: CTATGGGTCTGGTTATGGAA	(GGA)_11_	101–161	55	Cytosolic 5′‐nucleotidase 3, transcript variant X5	MG682122
	R: CTCCCAGCCAATATCTCAG					
TR31156	F: CTCTGCCTCTTTCTCTTCTCT	(AG)_14_	117–155	55	Putative glycine‐rich cell wall	MG682123
	R: ATCCCCCTACTTTCTTGC					
TR31399	F: GAAGAACAAGAAGGGCAAG	(AG)_20_	117–167	55	Structural protein 1	MG682124
	R: CAACACTAACACCACCAGATT					
TR31622	F: AAGCCAGAACCCTTATCTCTA	(AAC)_5_	125–179	55	Unknown	MG682125
	R: GCCTGAATTGAGTGAACTGTA					
TR32795	F: CAAGTTGATTTCTTCCTCTCC	(AGA)_13_	119–155	55	Peamaclein‐like	MG682126
	R: TATAACGCCTCTCTCTGTTGA					
TR33107	F: AAAACAGAGAGGACGATGG	(TTGT)_6_	139–171	55	BEL1‐like homeodomain protein 9, transcript variant X1	MG682127
	R: GCTCTAGGTGTTGGGTTAAAT					
TR35128	F: GCCTGAATTGAGTGAACTGTA	(TTG)_8_	129–181	56	Glutamine–fructose‐6‐phosphateaminotransferase [isomerizing] 2	MG682128
	R: AAGCCAGAACCCTTATCTCTA					
TR35536	F: CTCTTGTGTTCATCGGCTAC	(TGA)_6_	153–175	55	Calcium‐dependent protein kinase 13	MG682129
	R: ATCTCACCCGTCTCTTTATTC					
TR37605	F: CTTTCTAACCCCTCTGTGTCT	(TC)_21_	117–153	55	BEL1‐like homeodomain protein 9, transcript variant X2	MG682130
	R: TATGAACAGCTTACCTTCGTC					
TR37669	F: CCTCTTTGATCTCCTTCCATA	(TCT)_8_	141–181	55	Glutamate synthase [NADH], amyloplastic, transcript variant X3	MG682131
	R: GAAGACGACCTTTTTCATACC					
TR37811	F: GATCAAGGAGGAAGACAAAGT	(AG)_26_	105–145	55	Heterogeneous nuclear ribonucleoprotein 1	MG682132
	R: GCAGAGAGAGAGAAAGAGGAA					
TR38169	F: GATCATGAAGTTGCAGAAGAG	(TGG)_10_	143–185	56	Probable WRKY transcription factor 12	MG682133
	R: ATGTGCCCTTCCTGTGAG					
TR38361	F: GGATGTGCTTTACTGGAGAA	(TGA)_8_	127–155	56	Probable phospholipid‐transporting ATPase	MG682134
	R: CTCCTTCTAATCCCTTCAGC					
TR41171	F: TAGAAACTATGTGCGTGTGTG	(GA)_15_	123–175	55	4, transcript variant X2	MG682135
	R: CTTCAGGGAGGCTATAAATTC					
TR42254	F: ATTGCTTGCTCTGTCTCTGTA	(AAG)_5_	141–181	55	Homeobox‐leucine zipper protein HOX11‐like, transcript variant X2	MG682136
	R: CTTATTACCCACTACCCCAAC					
TR42785	F: TCTCTGAAATTCTCTCTGCTC	(GTGC)_6_	117–147	55	Formin‐like protein 4	MG682137
	R: TCTTTCTTCCTCCTCTCTCAC					
TR46482	F: CCACCTTCTTATAGCGACTG	(TATG)_6_	133–193	55	Probable E3 ubiquitin‐protein ligase RZFP34, transcript variant X4	MG682138
	R: AAGTACGATACAAGCACTCCA					
TR47534	F: CGATCTCCTTCCAGACCT	(GTG)_6_	119–157	55	Auxin efflux carrier component 1	MG682139
	R: CGACATCCTCAAGAACATCTA					
TR47552	F: ACAATCTCTTCGTCTCTGGTT	(AG)_19_	121–177	54	UDP‐glycosyltransferase 76E2‐like, transcript variant X2	MG682140
	R: GCTCCAGTGTACGTTGTAAG					
TR50321	F: AAGACCACTCACAACTCCAC	(CTT)_8_	263–293	55	Cyclic nucleotide‐gated ion channel 1‐like, transcript variant X1	MG682141
	R: CTTTGAAACACTGTTCTGGTC					
TR52495	F: TGATGAGCTTGCCATTCT	(GAA)_8_	147–173	55	G‐box‐binding factor 4, mRNA	MG682142
	R: CTTCTTGTCCGCTAATTCAC					

Note

*T*_a_ = annealing temperature.

A total of 51 primer sets produced consistent amplifications in all samples and were polymorphic (Table 1). These 51 loci had high numbers of effective alleles and high levels of observed and expected heterozygosity, ranging from seven to 11, 0.545 to 1.000, and 0.600 to 0.989, respectively (Table 2). Cross‐amplification of these 51 primer sets was tested for in *P. triloba* Lindl., *P. davidiana* (Carrière) Franch., *P. persica* (L.) Batsch, *P. cerasifera* Ehrh., and *P. serrulata* Lindl.; most primers amplified in these five species except *P. triloba* (*P. triloba*: 35/51; *P. davidiana*: 43/51; *P. persica*: 46/51; *P. cerasifera*: 45/51; *P. serrulata*: 41/51; Table 3).

**Table 2:** Genetic diversity of 51 microsatellite loci in three populations of *Prunus mongolica*.^a^

Locus	Wulanchabu population (*n* = 10)			Chifeng population (*n* = 10)			Eerduosi population (*n* = 12)		
	*A*_e_	*H*_o_	*H*_e_	*A*_e_	*H*_o_	*H*_e_	*A*_e_	*H*_o_	*H*_e_
TR3320	8.500	0.889	0.922	8.500	0.889	0.869	9.000	1.000	0.925
TR5622	11.000	1.000	0.928	10.500	1.000	0.963	9.500	0.833	0.880
TR9296	7.000	1.000	0.967	8.000	1.000	0.970	7.000	1.000	0.925
TR10339	11.000	1.000	0.900	11.000	1.000	0.832	10.000	1.000	0.935
TR10936	10.500	1.000	0.922	10.000	1.000	0.889	9.500	0.909	0.926
TR12256	10.000	0.889	0.837	9.500	0.800	0.600	9.500	0.800	0.847
TR12298	11.000	1.000	0.941	10.500	1.000	0.858	9.500	1.000	0.906
TR12728	10.000	1.000	0.948	10.000	1.000	0.954	9.000	1.000	0.874
TR14394	11.000	0.778	0.895	10.500	0.900	0.758	9.500	0.750	0.699
TR16484	10.500	1.000	0.908	10.000	1.000	0.921	9.500	1.000	0.909
TR17663	10.500	1.000	0.980	10.500	1.000	0.961	9.000	1.000	0.960
TR17745	11.000	1.000	0.974	10.500	1.000	0.953	9.500	1.000	0.928
TR18640	10.500	1.000	0.989	10.500	1.000	0.953	10.000	1.000	0.939
TR18685	11.000	0.875	0.908	10.000	0.800	0.858	9.000	0.833	0.790
TR18807	9.500	1.000	0.942	10.500	1.000	0.967	9.000	1.000	0.939
TR19174	11.000	0.889	0.810	10.500	1.000	0.853	9.500	1.000	0.938
TR19201	11.000	1.000	0.858	11.000	0.900	0.884	10.000	0.917	0.888
TR20030	11.000	1.000	0.941	10.500	1.000	0.953	9.500	0.917	0.928
TR20374	10.500	1.000	0.974	10.000	1.000	0.963	9.500	1.000	0.952
TR21328	11.000	1.000	0.963	11.000	1.000	0.958	10.000	1.000	0.960
TR21918	11.000	1.000	0.954	10.500	1.000	0.921	9.500	1.000	0.960
TR22071	11.000	0.889	0.817	10.500	0.900	0.942	9.500	0.750	0.928
TR23600	10.500	1.000	0.908	10.500	1.000	0.935	9.000	1.000	0.949
TR24122	9.000	1.000	0.922	8.500	0.900	0.937	9.500	1.000	0.925
TR25043	10.500	0.778	0.882	10.000	0.600	0.705	9.500	0.727	0.870
TR25166	11.000	1.000	0.915	10.500	1.000	0.963	9.500	1.000	0.920
TR25414	10.500	1.000	0.733	9.500	1.000	0.832	9.000	0.889	0.771
TR26908	10.000	0.900	0.884	10.500	0.889	0.791	9.500	0.909	0.905
TR27527	11.000	0.900	0.863	11.000	0.900	0.837	10.000	0.833	0.920
TR29327	10.000	1.000	0.928	10.000	1.000	0.876	9.000	1.000	0.952
TR30962	9.500	1.000	0.867	9.000	1.000	0.935	8.500	1.000	0.932
TR31156	10.000	0.889	0.974	10.000	1.000	0.915	9.000	1.000	0.931
TR31399	10.500	1.000	0.961	10.000	1.000	0.979	9.500	1.000	0.935
TR31622	11.000	0.800	0.884	11.000	1.000	0.916	10.000	0.833	0.851
TR32795	11.000	0.900	0.921	11.000	1.000	0.926	10.000	0.917	0.891
TR33107	11.000	0.900	0.911	11.000	0.900	0.874	10.000	0.833	0.909
TR35128	11.000	0.778	0.817	10.500	1.000	0.900	9.500	0.750	0.855
TR35536	10.500	0.778	0.882	10.000	0.900	0.837	9.500	0.909	0.900
TR37605	11.000	0.900	0.926	11.000	1.000	0.895	10.000	1.000	0.935
TR37669	10.500	0.889	0.869	10.500	0.889	0.889	9.000	1.000	0.899
TR37811	10.500	1.000	0.935	10.500	1.000	0.941	9.000	1.000	0.957
TR38169	10.500	1.000	0.941	10.500	1.000	0.961	9.000	0.917	0.935
TR38361	10.000	1.000	0.858	10.500	1.000	0.778	9.500	1.000	0.840
TR41171	10.500	1.000	0.967	10.000	1.000	0.980	8.500	1.000	0.931
TR42254	11.000	0.900	0.921	11.000	0.800	0.921	10.000	0.583	0.851
TR42785	10.000	0.750	0.833	10.000	0.875	0.867	8.000	0.833	0.866
TR46482	9.500	1.000	0.941	10.500	1.000	0.923	8.000	0.833	0.848
TR47534	9.000	1.000	0.817	8.500	0.778	0.680	8.500	0.556	0.804
TR47552	10.000	1.000	0.967	10.000	1.000	0.941	9.000	1.000	0.948
TR50321	9.000	0.800	0.874	10.500	0.857	0.868	8.500	0.545	0.827
TR52495	9.500	0.556	0.843	9.500	1.000	0.902	9.000	0.800	0.805

Note

*A*_e_ *=* number of effective alleles; *H*_e_ = expected heterozygosity; *H*_o_ = observed heterozygosity.

a Locality and voucher information are provided in Appendix 1.

**Table 3:** Cross‐amplification results for the 51 microsatellite loci developed in *Prunus mongolica* in five *Prunus* species.^a^^,^ b

Locus	*P. triloba*	*P. davidiana*	*P. persica*	*P. cerasifera*	*P. serrulata*
TR10339	—	163–195	115–147	133–171	117–151
TR10936	—	137–149	119–146	125–167	149–161
TR12298	—	103–115	—	132–144	105–121
TR12728	—	110–122	105–117	105–127	120–132
TR16484	—	153–165	143–155	133–145	113–125
TR17663	—	131–140	130–142	—	120–129
TR17745	120–126	110–122	123–135	133–145	125–137
TR18640	135–147	153–159	133–145	131–143	—
TR18807	140–149	143–155	133–145	113–119	131–143
TR20374	115–127	151–163	157–169	153–165	151–159
TR21328	111–131	115–136	113–125	117–129	—
TR21918	101–121	110–130	115–135	118–138	111–123
TR23600	—	—	80–100	91–101	—
TR24122	—	—	130–150	139–159	133–153
TR3320	—	—	—	121–133	—
TR27527	140–149	145–160	132–144	141–153	135–147
TR29327	133–145	135–147	130–142	132–138	—
TR31156	—	—	—	—	141–161
TR31399	131–151	111–131	143–163	141–161	—
TR37605	125–145	126–146	121–141	122–142	123–143
TR37811	131–161	121–151	111–141	146–176	132–162
TR41171	131–191	112–142	123–153	119–149	145–175
TR47552	121–141	122–142	111–131	123–143	101–131
TR26908	113–129	133–149	113–125	115–127	131–143
TR30962	111–127	131–143	127–139	123–139	135–147
TR32795	125–137	123–126	147–159	131–161	126–138
TR19174	103–123	121–133	116–128	113–129	132–144
TR19201	142–154	131–161	125–137	126–138	141–171
TR20030	115–135	117–139	123–135	134–146	137–149
TR25166	113–125	132–144	115–135	162–182	133–145
TR31622	151–163	131–143	133–163	142–154	125–137
TR35128	117–129	146–158	135–155	132–144	172–184
TR35536	137–157	117–129	153–165	135–155	127–167
TR37669	123–138	121–133	125–137	122–134	115–127
TR38169	—	—	125–137	—	—
TR38361	133–149	131–147	115–127	124–136	125–137
TR42254	122–134	117–129	115–127	123–135	116–128
TR47534	123–135	115–127	132–144	124–136	125–137
TR52495	117–129	112–124	113–125	117–129	123–135
TR5622	121–151	124–136	142–154	141–153	123–135
TR12256	—	103–115	105–117	113–133	132–152
TR14394	143–155	124–136	121–130	123–135	141–153
TR22071	122–155	114–126	142–154	121–133	113–125
TR50321	—	—	—	123–143	114–124
TR18685	113–125	121–133	135–147	123–135	141–153
TR25043	—	117–137	132–144	124–136	—
TR25414	133–145	123–139	121–133	142–158	152–164
TR33107	121–133	143–155	117–139	—	113–128
TR42785	—	—	—	—	—
TR46482	253–265	221–236	232–244	—	215–227
TR9296	—	—	141–156	122–134	—

Note

— = unsuccessful amplification.

a Locality and voucher information are provided in Appendix 1.

b *N* = 5 for all populations.

## CONCLUSIONS

In our study, a total of 19,498 SSRs were identified from 15,837 contigs, and 6069 microsatellite primers were designed. Among the identified SSR loci, 51 microsatellite primer pairs were polymorphic. The 51 novel microsatellite markers can be used to study the genetic diversity, landscape genetics, and phylogeography of the species. The microsatellite primers can also be used to genotype congeneric species (*P. triloba*,* P. davidiana*,* P. persica*,* P. cerasifera*, and *P. serrulata*).

## APPENDIX 1 Voucher information for *Prunus* species used in this study.

**Table:**

Species	*n*	Collection locality	Geographic coordinates	Voucher ID^a^	Collector
*Prunus mongolica* Maxim.	10	Wulanchabu, Inner Mongolia, China	41°56′N, 110°28′E	201607‐Pmon‐popI‐AHB	Zhanyuan Lu
	10	Chifeng, Inner Mongolia, China	43°23′N, 118°40′E	201607‐Pmon‐popII‐AHB	Zhanyuan Lu
	12	Eerduosi, Inner Mongolia, China	39°35′N, 106°46′E	201607‐ Pmon‐popIII‐AHB	Zhanyuan Lu
*P. triloba* Lindl.	5	Hefei, Anhui, China	31°52′N, 117°11′E	201705‐Ptri‐AHB	Xin Hong
*P. davidiana* (Carrière) Franch.	5	Hefei, Anhui, China	31°52′N, 117°11′E	201705‐Pdav‐AHB	Xin Hong
*P. persica* (L.) Batsch	5	Wuhan, Hubei, China	30°32′N, 114°24′E	201705‐Pper‐AHB	Xin Hong
*P. cerasifera* Ehrh.	5	Hefei, Anhui, China	31°52′N, 117°11′E	201705‐Pcer‐AHB	Xin Hong
*P. serrulata* Lindl.	5	Hefei, Anhui, China	31°52′N, 117°11′E	201705‐Pser‐AHB	Xin Hong

Note

*n* = sample size.

a Vouchers are deposited at the herbarium of Anhui University (ANU), Hefei, Anhui Province, China.
